# Artificial Intelligence-Driven Cost Savings from Emergency Department Chest Pain Patient Evaluation: A Monte Carlo Simulation

**DOI:** 10.5811/westjem.56973

**Published:** 2026-07-28

**Authors:** Christopher W. Baugh, Andrew Luo, Christopher Zeuthen, Kian D. Samadian, Ricardo E. De Armas, Michael Senter-Zapata, Michael J. Zellweger, Hans-Peter Brunner-LaRocca

**Affiliations:** * Harvard Medical School, Brigham and Women’s Hospital, Department of Emergency Medicine, Boston, Massachusetts; † Harvard Medical School, Massachusetts General Hospital and Brigham and Women’s Hospital, Department of Emergency Medicine, Boston, Massachusetts; ‡ Wake Forest University School of Medicine, Winston-Salem, North Carolina; § Massachusetts General Hospital, Department of Cardiology, Boston, Massachusetts; ¶ Maastricht University Medical Centre, Department of Cardiology, Maastricht, The Netherlands; || Massachusetts General Hospital, Department of Gastroenterology, Boston, Massachusetts; # University of Basel, University Hospital Basel, Department of Cardiology, Basel, Switzerland

## Abstract

**Introduction:**

Chest pain patients presenting to the emergency department (ED) often require risk stratification through stress testing, which may necessitate hospitalization. This practice contributes to ED crowding by occupying beds with low-risk patients who may derive limited benefit from testing, thereby increasing bed occupancy, prolonging ED boarding times, amplifying crowding, and resource strain. Use of an artificial intelligence (AI)-driven clinical decision support tool improves the detection of obstructive coronary artery disease and focuses diagnostic testing on those most likely to benefit.

**Methods:**

We created a Monte Carlo simulation informed by available published inputs and ran 1,000 trials to estimate the national impact of using an AI-driven decision-support tool to reduce avoidable downstream cardiac testing among eligible U.S. ED patients with chest pain. Model inputs included patient demographics, chest pain history, electrocardiogram (ECG) findings, medication use, and lab values (including some nonroutine tests). Our primary outcome was U.S. annual cost savings from reclassifying eligible ED patients using an AI-driven decision-support tool. Secondary outcomes included reductions in short-stay hospitalizations, cancer cases, and cancer deaths due to averted radiation exposure.

**Results:**

Universal adoption of an AI-driven decision-support tool was estimated to save a mean (standard deviation) of $675 million ($340 million) by avoiding 688,000 (147,000) downstream cardiac diagnostic tests. This resulted in annual decreases of 537,000 (115,000) hospitalizations, 8.30 million (1.96 million) bed hours, 490 (150) new cancer diagnoses, and 250 (80) cancer deaths.

**Conclusion:**

Assuming similar recategorization of patients seen in a European outpatient cohort, widespread adoption of an AI-driven clinical decision-support tool to evaluate U.S. ED patients with suspected obstructive coronary disease could yield substantial benefits by reducing avoidable downstream cardiac testing. By accurately identifying patients who do not require urgent diagnostics, this approach could help mitigate crowding, relieve resource strain, and improve patient flow—supporting a more efficient emergency care system.

## INTRODUCTION

Chest pain is the second leading cause of emergency department (ED) visits in the United States (U.S.), accounting for approximately 7.8 million encounters annually, or 5.5% of all ED visits.^1^ Traditional diagnostic tools, including high-sensitivity troponin (hsTn) tests and electrocardiograms (ECG), detect acute myocardial infarction but cannot diagnose obstructive coronary artery disease (CAD) without myocardial injury. Consequently, many patients undergo prolonged ED observation or hospitalization for additional cardiac testing, such as coronary computed tomography angiography (CCTA) and stress testing (ST). Among low-risk patients, these tests consume bed capacity, increase healthcare costs, and cause unnecessary radiation exposure without improving diagnostic yield.^2^,^3^

Several clinical scores and accelerated diagnostic protocols have been developed to improve risk stratification for chest pain.^4^ The HEART score (history, ECG, age, risk factors, and initial troponin) is widely adopted in U.S. EDs due to its high sensitivity for acute coronary syndrome (ACS) and its low adverse-event rate in low-risk patients.^5^–^14^ However, its utility for assessing CAD prevalence is limited, particularly in the absence of acute myocardial injury. Moreover, the HEART score prioritizes sensitivity over specificity (pooled sensitivity of 96% and specificity of ~50% predicting major adverse cardiac events [MACE]), resulting in a high rate of false positives and downstream testing.^15^,^16^ Other models, such as the Diamond Forrester and Duke clinical scores, are recommended by the American Heart Association and American College of Cardiology for estimating CAD prevalence,^17^–^20^ but they often overestimate risk and are rarely used in the ED setting.^19^,^21^,^22^ In 2019, the European Society of Cardiology introduced the CAD2 model to improve CAD risk prediction.^23^,^24^ While CAD2 demonstrates enhanced accuracy, its performance in diverse populations has been limited, and its complexity hinders its practical utility in ED workflows.^24^,^25^

These limitations highlight a gap in current ED chest pain evaluation: existing tools are designed to avoid missed events rather than efficiently identify patients who could safely forgo additional testing. Recent studies demonstrate that moderate-risk patients with CAD are at a higher risk of a 30-day rate of MACE than those without CAD, further highlighting the need for improved predictive models.^27^ In recent years, advances in machine learning have led to the development of multiple artificial intelligence (AI)–based models aimed at improving CAD detection and risk stratification using routinely available clinical data. These approaches extend beyond traditional rule-based scores by integrating high-dimensional inputs, including lab values, ECG features, and clinical variables, to generate individualized risk predictions. Several studies have demonstrated that machine learning models can outperform conventional risk scores in predicting obstructive CAD and MACE.^28^–^30^

**Population Health Research Capsule**

What do we already know about this issue?

*Emergency department chest pain evaluation often leads to avoidable cardiac testing for low-risk patients, increasing hospitalizations and healthcare costs*.

What was the research question?

*Could an artificial intelligence (AI) decision-support tool reduce avoidable cardiac testing and costs in U.S. emergency department chest pain patients?*

What was the major finding of the study?

*A Monte Carlo simulation showed mean (SD) annual savings of $675 million ($340 million), a 17.6% reduction in total spending from 688,000 (147,000) tests averted*.

How does this improve population health?

*Risk stratification guided by AI could prevent 537,000 U.S. hospitalizations, reduce crowding by 8.3 million bed hours, and avert ~490 cancer cases and 250 deaths annually*.

One such model, Cardio Explorer (Exploris Health AG, Zürich, Switzerland), is an AI tool created via a memetic pattern-based algorithm that integrates multiple analytical methods to evaluate CAD risk as an integrated tool in the electronic health record (EHR) using readily available patient data, supplemented by additional blood tests (no additional inputs are used by Cardio Explorer beyond those listed in Figure 1).^31^,^32^ Validated across low- to high-risk European populations and Conformité Européenne approved for healthcare safety, Cardio Explorer demonstrates superior capability for identifying clinically significant CAD compared to traditional models.^31^,^32^ For example, in one validation study of 696 Dutch patients referred for outpatient cardiac testing, Cardio Explorer had a negative predictive value (NPV) of 95.8%, sensitivity of 82.3%, specificity of 77.4%, and area under the receiving operating curve of 0.87 against a gold standard of cardiac catheterization, CCTA, or nuclear perfusion ST.^31^ The Cardio Explorer NPV was comparable to other risk scores, while categorizing more than twice as many patients as very low or low risk (84.4% versus 40.2% and 44.5% for Diamond Forrester and CAD2 models, respectively). These results indicate good overall discriminatory performance and the ability to classify a larger proportion of patients as low risk compared with traditional approaches.

Existing risk stratification tools, such as the HEART score, rely on threshold-based categorizations, often leading to additional testing and observational management. For example, a low-risk 45-year-old with nonexertional chest discomfort may be classified as intermediate risk, prompting additional testing despite a low likelihood of disease, whereas a 62-year-old with exertional chest pain may not meet categorical thresholds despite higher underlying risk. Given the complexity of chest pain evaluation, a clinical decision support tool such as Cardio Explorer, which provides risk along a continuous spectrum, may enable more precise stratification and more efficient use of resources.

While a prospective U.S. validation study is planned, in this current study we conducted a cost-savings modeling analysis of implementing Cardio Explorer for patients without known CAD presenting to the ED with chest pain, using the HEART pathway as the comparative model. We evaluate Cardio Explorer’s potential to minimize unnecessary downstream cardiac testing, reduce radiation exposure, and provide a cost-effective approach to managing chest pain in the ED.

## METHODS

We developed a Monte Carlo simulation model to estimate annual national healthcare cost savings, cardiac diagnostic tests averted, reductions in short-stay hospitalizations, and radiation exposure prevented among eligible ED patients with chest pain undergoing Cardio Explorer evaluation. Monte Carlo simulations run many model iterations using random values selected from underlying data distributions that best represent each model input.^33^ The program averages results of each model iteration to display outcome variables as distributions with means and standard deviations, accounting for uncertainty inherent in the model inputs and assumptions. We repeated our analysis for common ED sizes (eg, 30,000, 60,000, and 90,000 annual adult visits) to illustrate institution-specific impact.

Monte Carlo simulations have been used in medical research, including evaluating the national cost savings from dedicated observation units.^34^ We conducted a standard simulation using 1,000 iterations, with input parameters derived from the most recent and highest-quality data available via a literature search. We followed the RECORDS checklist for Monte Carlo studies involving medical physics, given our radiation exposure secondary outcomes (see Online Supplement, Table 2).^35^ We analyzed publicly available data exempt from institutional review board evaluation. Our model and inputs are displayed in Figure 2 and Table 1.

We estimated input parameters using the most recent National Hospital Ambulatory Care Survey (NHAMCS) for adult U.S. ED patient volume.^36^ Next, we estimated the percentage undergoing downstream cardiac diagnostic testing for obstructive CAD within one week of the index visit, as determined in a prior MarketScan commercial claims analysis.^2^ This analysis also details the relative frequency of cardiac diagnostic options, including exercise tolerance testing, nuclear perfusion testing, stress echocardiography, and CCTA. To examine the expected change in ST and CCTA from Cardio Explorer use, we used the current proportion of the U.S. ED population with chest pain. categorized as low risk, as determined by a recent U.S. study that used the HEART pathway (38.4% low risk).^12^ While the mean age and incidence of hyperlipidemia were higher in the European Cardio Explorer study (65.6 versus 54.0 years; 53.9% versus 43.7%), body mass index > 30 and hypertension rates were higher in the U.S. HEART pathway study (47.4% versus 20.3%; 65.4% versus 45.7%). Next, we examined patient reclassifications from “medium risk,” “high risk,” and “very high risk” to “low risk” or “very low risk” in Cardio Explorer studies (67.7% low risk).^31^ Assuming a similar recategorization among U.S. ED patients, we modeled a net reduction in downstream testing for those reclassified as “low risk” or “very low risk,” resulting in approximately a 50% reduction, from 18.5% to 9.7%.

We used Medicare payments as a proxy for hospital and physician service costs, consistent with prior cost-savings analyses and avoiding the arbitrary and variable values associated with charges and payer-specific payments for the same services, respectively.^50^ In the base-case model, we assumed a $50 cost per Cardio Explorer visit, reflecting common costs for similar tools in a previous systematic review.^73^ We did not separately include labor costs, as they are embedded in the Centers for Medicare & Medicaid Services (CMS) facility payment bundles. We accounted for lab test costs required for the Cardio Explorer risk assessment, which are not typically included in an ED chest pain visit, excluding cases with obvious ST-segment elevation myocardial infarction on arrival or cases in which ACS was never in the differential diagnosis.^37^,^38^

We also estimated the percentage of visits with cardiac risk stratification testing as the rate-limiting step for discharge, allowing us to estimate the changes in hospitalizations and bed hours averted by reducing ST and CCTA (defined as the cumulative time patients occupy beds in the ED, effectively a proxy for length of stay). To account for differences in patient populations and clinical culture between the U.S. and Europe, we assumed a maximum adoption of 90% in the U.S. in our base case. Finally, we leveraged prior radiation exposure harm data (eg, 800 excess solid cancer cases per 100,000 males from 100-millisievert exposure) and expected test radiation (eg, 10-millisievert mSv for single photon emission computed tomography perfusion ST) to estimate reductions in cancer cases and deaths from avoided tests.^49^

We performed a sensitivity analysis examining the impact of varying key assumptions with the greatest uncertainty: the percentage of visits in which the clinician followed Cardio Explorer recommendations despite practice inertia or alternative risk-stratification models suggesting testing was indicated, and the percentage of visits in which cardiac diagnostics were the rate-limiting step for discharge.

### Statistical Analysis

We used Crystal Ball release 11.1.2.4 (Oracle Corporation, Austin, TX) for analysis. Based on the reported standard deviations (SD), we assumed a normal distribution for all inputs. Unless otherwise specified, we assumed a 10% relative SD for input estimates, with bounds of 0% and 100% for percentage values. For length-of-stay inputs, we assumed Poisson distributions, the most suitable for this dataset.^51^ For inputs with interquartile ranges or 95% confidence intervals, we assumed BetaPERT (beta program and evaluation review technique) distributions–smooth distributions characterized by minimum, most likely, and maximum values.^52^,^53^ Accordingly, they are most appropriate for describing variables with a reported range of values.

## RESULTS

### Main Model Outputs

We found that using Cardio Explorer to determine patient eligibility for downstream cardiac diagnostics resulted in a mean U.S. national annual savings (SD) of $675 million ($340 million), representing a 17.6% reduction from the current state, by avoiding 688,000 (147,000) cardiac diagnostic tests. In Figures 3, 4a, 4b, 5a, and 5b, we present the resulting national annual cumulative decreases of 537,000 (115,000) hospitalizations, 8.30 million (1.96 million) bed hours, 490 (150) new cancer diagnoses, and 250 (80) new cancer deaths attributed to radiation exposure from avoidable cardiac diagnostics.

To examine the facility-level impact of Cardio Explorer in this patient population, we also estimated the annual cost savings for an ED with various common annual visit volumes using the other base-case model assumptions. At 30,000 annual adult visits, the cost savings would be $115,000 ($65,000) with 1,440 (350) fewer bed hours and 93 (20) avoided hospitalizations. At 60,000 annual adult visits, the cost savings would be $228,000 ($129,000) with 2,900 (700) fewer bed hours and 190 (40) avoided hospitalizations. Finally, at 90,000 annual adult visits, the cost savings would be $336,000 ($201,000) with 4,330 (1,080) fewer bed hours and 280 (60) avoided hospitalizations.

### Sensitivity Analysis

We varied the assumption of a 95% Cardio Explorer compliance rate to examine the impact on our primary outcome, cost savings, by avoiding further cardiac testing. Reducing this value to 80% reduces the cost savings to $439 million ($296 million). Further reducing it to 70% results in cost savings of $278 million ($255 million). Lastly, varying the assumption that cardiac diagnostics are the rate-limiting step for hospital discharge in 90% of cases to 70% reduces the national cost savings to $271 million ($363 million). Lastly, in Table 2, we illustrate the impact of various prices per visit for Cardio Explorer on national and department-level annual cost savings.

## DISCUSSION

This study evaluated the potential benefits of incorporating Cardio Explorer, a novel clinical decision support tool, into the ED evaluation of patients with chest pain and concern for obstructive CAD. While our analysis demonstrates that this specific tool could generate $675 million in annual national cost savings and incremental reduction in radiation-associated cancer cases and deaths, primarily by reducing avoidable cardiac diagnostic testing and associated hospitalizations, the broader implication is more fundamental in that ED chest pain evaluation is entering an era of AI-augmented risk stratification. Rather than viewing Cardio Explorer as a standalone innovation, it should be understood as part of a rapidly expanding ecosystem of AI–enabled diagnostic tools designed to address uncertainty in acute cardiovascular care.

Cardio Explorer should be viewed within the context of evolving U.S. ED practice patterns, where hsTn and AI-driven clinical decision support tools are becoming increasingly prevalent.^54^ In Europe, hsTn has been widely adopted, whereas only about one-third of U.S. hospitals have converted from conventional troponin assays.^55^,^56^ Recent analyses of U.S. hospitals adopting hsTn accelerated diagnostic protocols show significant reductions in downstream cardiac testing and hospitalizations with substantial cost savings.^57^–^59^ Cardio Explorer is a rational evolution and companion to these protocols, further reducing avoidable cardiac testing and hospitalizations.

A persistent challenge in chest pain management is the large proportion of patients, over 50% in some studies, who fall into the “observation zone,” not meeting discharge or admission criteria and creating clinician uncertainty about discharge without additional testing.^60^,^61^ Cardio Explorer addresses this ambiguity by reclassifying a substantial part of these indeterminate-risk patients into more definitive categories of “very low,” “low,” “high,” or “very high” risk.^26^ This may improve clinicians’ confidence in discharge decisions and yield additional savings as hsTn testing prevalence increases and clinicians contend with more cases of troponin levels of ambiguous clinical significance. Additionally, Cardio Explorer may address emergency physicians’ historical tendency to recommend downstream cardiac testing for patients with possible unstable angina, a component of ACS characterized by the absence of troponin patterns indicating myocardial injury or ST-segment elevations suggestive of STEMI.^63^ Obstructive CAD without ACS is often considered a chronic condition managed by primary care physicians and cardiologists on an elective basis following ED visits. However, identifying obstructive CAD in ED chest pain. patients, whether or not ACS accompanies it, is crucial for optimizing both immediate and longer-term clinical outcomes.

Current cardiac diagnostic pathways lead to prolonged hospital stays even when the risk is unclear or even low. As over 90% of EDs routinely report crowded conditions, reducing bed hours is one of the most cost-effective ways to increase capacity, improve safety, and boost financial performance without adding new beds or staff. For example, one inner-city teaching hospital model estimated that a one-hour reduction in ED boarding could increase daily hospital revenue by approximately $9,693 to $13,298–equivalent to several million dollars annually.^64^ This demonstrates how improving patient flow enhances both care and hospital financial performance.^65^

Cardio Explorer not only improves identification of truly low-risk patients but also appropriately up-classifies a small subset into higher risk categories—individuals that conventional methods may miss. In the validation study, Cardio Explorer categorized 8.5% of patients as “very high risk,” compared to 0% and 1.7% in the comparator risk assessments.^31^ This up-triaging does not significantly affect index visit costs but may avoid downstream expenses from delayed disease recognition and serves as an important quality and safety mechanism. Currently, these patients are considered acceptable misses due to imperfect risk-stratification tools and the low yield of universal cardiac testing.^66^ Cardio Explorer enables a more personalized approach, matching each patient’s risk to the diagnostic strategy. In recent years, the promise of precision medicine has been limited primarily to managing chronic diseases and developing targeted therapies.^67^ Tools such as Cardio Explorer are advancing precision medicine into the acute care setting. In the ED context, this represents a novel application leveraging real-time biomarkers and clinical data to support individualized triage decisions at scale.

The additional blood tests required by Cardio Explorer could be feasibly added to chest pain order sets with minimal additional effort. Electronic decision support is increasingly used in the clinical space; nearly all EDs now use an EHR, making integration feasible with current technology.^68^,^69^ With proper EHR configuration, Cardio Explorer could be embedded as a click-through module or automated through order sets with minimal staff orientation. However, real-world implementation may require attention to clinician training, workflow integration, and addressing potential skepticism or alarm fatigue. We must also evaluate usability, physician trust, and the impact on decision-making to ensure sustainable integration.

Finally, the projected reductions in radiation-associated cancer cases and deaths should be interpreted cautiously. Cancer risk estimates from low-dose diagnostic imaging remain debated, as commonly used models extrapolate risk from higher dose exposures and may overestimate harm at low doses due to uncertainty in dose–response relationships and confounding.^44^,^71^ While these limitations preclude precise quantification of long-term cancer risk, reducing low-yield imaging remains a widely accepted patient-safety objective. Accordingly, our estimates should be viewed as directional indicators of potential harm reduction rather than definitive predictions of cancer prevention.

Historical risk-stratification models needed to be simple enough for busy clinicians to use in real time, necessitating a limited number of variables and categorizing patients into broad risk groups. Small changes in risk could move a patient from one risk category to another, leading to vastly different management recommendations. For example, a single point difference in the HEART pathway could shift patients from low to intermediate risk, altering the recommendation from discharge without testing to hospitalization for cardiac testing.^68^ Such systems can lead to both overuse and underuse of testing depending on arbitrary cutoffs. In contrast, Cardio Explorer employs a continuous, data-rich risk spectrum that reduces reliance on rigid thresholds and better reflects the complex cardiac risk profiles of real-world patients at low marginal cost. While our primary focus is cost savings and reduced radiation exposure, we must acknowledge the potential trade-offs in missed diagnoses if diagnostic imaging is reduced. Cardio Explorer has demonstrated high sensitivity for ischemia prediction in validation studies among intermediate- to high-risk patients.^69^ Nonetheless, with a sensitivity < 100%, any shift in diagnostic protocols must be accompanied by careful safety monitoring. Future studies should evaluate clinical outcomes, such as 30-day rates of MACE, to ensure that testing reductions do not compromise the detection of high risk pathology.

Future innovations in ED chest pain evaluation include further refining tools like Cardio Explorer to increase the proportion of patients deemed “very low” and “low” risk, enabling more selective downstream cardiac testing and reducing harm from missed CAD diagnoses. Next steps include validating Cardio Explorer in a U.S.-based ED population. Our analysis could be updated with data from this study to validate the assumptions used. Nevertheless, the underlying physiological patterns used by Cardio Explorer are likely conserved across populations, and the model’s adaptability suggests strong generalizability potential once formally tested in the U.S. The significance of certain blood tests (eg, uric acid, amylase) in the Cardio Explorer risk assessment has unclear underpinnings in obstructive CAD pathophysiology based on our historical understanding. Future investigations may better clarify these relationships, further enhancing detection and treatment approaches.

## LIMITATIONS

Our analysis was based on a simulation model and, thus, was limited by the accuracy of model inputs and structure. We employed Monte Carlo methods to account for uncertainty and used recent, widely accepted sources to inform model variables. Due to the unavailability of more recent analyses, some of our references for model inputs were older and may not reflect current practice patterns. However, we made assumptions and adjustments when data were unavailable. Importantly, we did not account for the costs of EHR integration. However, our main model accounted for the laboratory test costs required by Cardio Explorer, which are not typically part of ED chest pain evaluations. Our analysis should thus inform decision-makers of the price they are willing to pay for this tool while still capturing significant savings and admission avoidance (Table 2).

In addition to the direct cost savings from reduced avoidable testing and hospitalizations, there are several potential benefits not explicitly captured in our model. For example, reclassification from low to high risk in a patient who would have otherwise been discharged could lead to revascularization in a patient who may have otherwise died without a timely, accurate diagnosis. Other investigations have used concepts such as quality-adjusted life years saved and benchmark financial assumptions to quantify the financial value of reduced mortality.^70^ Additionally, when appropriate, early discharge avoids unnecessary patient anxiety, missed work, gaps in caregiver coverage, and other indirect costs associated with hospitalization. Chest pain visits with ECG and troponin, performed to evaluate low-risk presentations where additional cardiac testing was previously not considered, would not be affected by Cardio Explorer, except in rare cases where a patient is unexpectedly classified as high-risk by the tool. Finally, a reduction in patient out-of-pocket expenses from reduced testing is another benefit not explicitly quantified in our model.

Additionally, the CAD rate among Cardio Explorer patients classified as “very low” or “low” was approximately 4.2%.^31^ These data were collected among European outpatients with chest pain referred for further testing. It is unclear whether our assumption that a U.S. ED population would experience the same risk profile recategorization observed is valid without confirmation. We also used data from prior HEART pathway studies, which are not universally followed in U.S. EDs and have been validated only for 30-day MACE, not CAD. Conversely, Cardio Explorer has been validated to predict occlusive CAD, not 30-day MACE. Given Cardio Explorer’s reported sensitivity, some patients classified as low risk may still be at risk for 30-day MACE. While the presence of occlusive CAD likely correlates with 30-day MACE among chest pain patients, further research is needed to study Cardo Explorer’s test characteristics with respect to this important clinical outcome.

Physicians in the U.S. may also be motivated by factors different from those of their European counterparts (eg, medicolegal risk), which may affect their willingness to avoid testing, thereby reducing expected cost savings from adopting this cardiac testing paradigm. Furthermore, patients with barriers to outpatient follow-up may be considered for additional diagnostics during the index visit, mitigating some of the cost savings identified in our primary analysis, as captured in our sensitivity analysis.

We represent cost savings as reduced avoidable healthcare expenses, which may be best realized in accountable care organizations or global budget frameworks, such as those in most European countries or Maryland. In a fee-for-service environment, hospitals may capture cost savings less directly and may experience revenue declines, negatively impacting net operating budgets. Nonetheless, the operational benefits of improved ED capacity, reduced crowding, and enhanced quality of care support the broader value of this tool even in traditional reimbursement environments. Hospitals at capacity, especially tertiary care centers, that regularly decline or delay transfer requests due to crowding, may particularly benefit from increased backfill of more complex patients and the associated higher payments. Prior investigations reveal that the opportunity cost of shifting patients to less resource-intensive plans of care (eg, inpatient to observation units) nearly doubles direct cost savings.^72^

Finally, although Exploris Health, the developer of Cardio Explorer, funded this study, the sponsor had no role in study design, model development, data analysis, interpretation of results, or manuscript preparation. Several authors have also disclosed financial relationships with the sponsor but were included due to their publication history and clinical expertise. We aimed to provide full transparency regarding these associations while explicitly detailing our methodology to enable reproducible results and mitigate bias.

## CONCLUSION

Our simulation model indicates that integrating a novel digital decision-support tool into ED chest pain evaluation could improve patient selection for downstream cardiac testing. Widespread adoption could lead to substantial national cost savings, reduce unnecessary short-stay hospitalizations, and lower radiation exposure, thereby preventing avoidable cancer cases and related mortality. Reduced ED bed-hours directly translate into decreased crowding and more efficient patient flow throughout the care continuum. Further research is warranted to validate its effectiveness in U.S. emergency care settings and to identify barriers to broader implementation.

## Supplementary Information



## Figures and Tables

**Figure 1:**
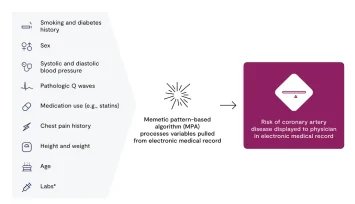
Illustration of an artificial intelligence-driven clinical decision tool for emergency department patients with chest pain. *Mean corpuscular hemoglobin concentration, white blood cells, urea, uric acid, troponin, glucose, total cholesterol, low-density lipoprotein cholesterol, high-density lipoprotein cholesterol, alanine aminotransferase, alkaline phosphatase, amylase, total protein, albumin, and bilirubin. See Online Supplement Table 1 for the medications considered by the AI tool.

**Figure 2a:**
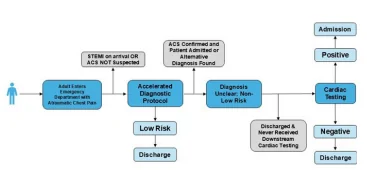
Current state patient flow model for emergency department chest pain visits. Accelerated diagnostic protocol for department-specific chest pain protocol specifying testing (e.g., electrocardiogram, troponin) and disposition options based on test results and risk stratification. *ACS*, acute coronary syndrome; *STEMI*, ST-segment elevation myocardial infarction.

**Figure 2b:**
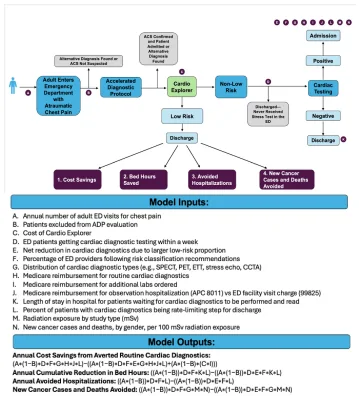
Patient flow model using the artificial intelligence-driven clinical decision tool Cardio Explorer for emergency department chest pain visits. *ACS*, acute coronary syndrome; *ADP*, accelerated diagnostic protocols; *APC*, ambulatory payment classification; *CCTA*, coronary computed tomography angiogram; *ED*, emergency department; *ETT*, exercise tolerance test; *mSv*, millisievert; *PET*, positron emission tomography; *SPECT*, single photon emission computed tomography.

**Figure 3:**
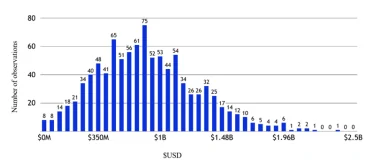
Annual national cost savings from Cardio Explorer use. Distributions reflect results from 1,000 Monte Carlo simulation iterations, revealing a mean of $675 million ($340 million). *B*, billion; *M*, million; *USD*, United States dollars.

**Figure 4a:**
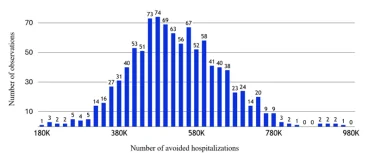
Annual national avoided hospitalizations from Cardio Explorer use. Distributions reflect results from 1,000 Monte Carlo simulation iterations, revealing a mean of 537,000 (115,000). *K*, thousand.

**Figure 4b:**
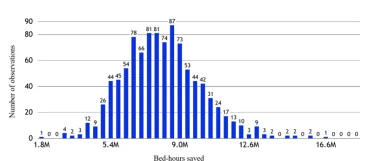
Annual national bed hours avoided from Cardio Explorer use. Distributions reflect results from 1,000 Monte Carlo simulation iterations, revealing a mean of 8.30 million (1.96 million). *M*, million.

**Figure 5:**
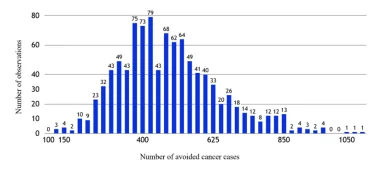

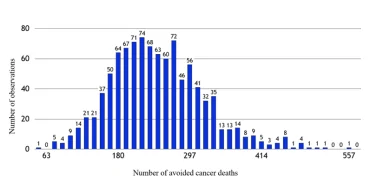
Annual reduction in radiation-exposure-related national cancer cases and deaths. **5a**. Avoided annual cancer cases from Cardio Explorer use. Distributions reflect results from 1,000 Monte Carlo simulation iterations, revealing a mean of 490 (150). **5b**. Annual avoided cancer deaths from Cardio Explorer use. Distributions reflect results from 1,000 Monte Carlo simulation iterations, revealing a mean of 250 (80).

**Table 1:** Model inputs for Monte Carlo simulation of Cardio Explorer use.

Input	Description	Estimate	SD or Interval	Distribution Type	Source and Reference
A	Annual number of ED chest pain visits in the US	8,354,000	826,000	Normal	2022 NHAMCS (United States)^36^
B	Patients excluded from ADP evaluation	16.5%	1.65%	Normal	2017 Multicenter STEMI Registry^37^ and 2025 Epic Cosmos Analysis (United States)^38^
C	Cost of Cardio Explorer	$50	NA	Author assumption	Author assumption; see Limitations and Online Supplement
D	Percentage of patients in “A” who receive further cardiac diagnostics within a week of the ED visit	18.5%	2.9%	Normal	2011 MarketScan Commercial Claims and Encounters data (United States)^2^
E	Percentage of patients receiving further cardiac diagnostics	9.7%	0.9%	Normal	Change from “B” above due to an increase in the “very low risk” and “low-risk” groups (Netherlands^31^; United States^39^)
F	Percentage of clinicians following advanced diagnostic protocol	95%	NA	NA	Author assumption; see Sensitivity Analysis
**Patients who go on to further cardiac diagnostics distributed as F.1–F.6**					
G.1	Percentage of conventional stress tests that are myocardial perfusion tests	64.8%	0.6%	Normal	2011 MarketScan Commercial Claims and Encounters data (United States)^2^
G.2	Percentage of perfusion tests that are SPECT	97.0%	9.7%	Normal	Medicare 2010–2019 Physician/Supplier Procedure Summary files (United States)^40^
G.3	Percentage of perfusion tests that are PET	3%	0.3%	Normal	Medicare 2010–2019 Physician/Supplier Procedure Summary files (United States)^40^
G.4	Percentage of conventional stress tests that are Stress Echo	12%	1.2%	Normal	2010–2017 National Emergency Database (United States)^41^
G.5	Percentage of conventional stress tests that are ETTs	14.2%	1.4%	Normal	2011 MarketScan Commercial Claims and Encounters data (United States)^2^
G.6	Percentage of cardiac diagnostic tests that are CCTA	7.4%	0.7%	Point estimate	2010–2017 National Emergency Database (United States)^41^
**Medicare reimbursement for cardiac testing by type and additional labs needed for Cardio Explorer**					
H.1	Medicare payment for perfusion stress/SPECT (facility)	Bundled in APC 8011	NA	Point estimate	2025 CMS Medicare Physician Fee Schedule (United States)^42^
H.2	Medicare payment for perfusion stress/SPECT (interpretation)	$62.55	NA	Point estimate	2025 CMS Medicare Physician Fee Schedule (United States)^42^
H.3	Medicare payment for PET (facility)	Bundled in APC 8011	NA	Point estimate	2025 CMS Medicare Physician Fee Schedule (United States)^42^
H.4	Medicare payment for PET (interpretation)	$82.55	NA	Point estimate	2025 CMS Medicare Physician Fee Schedule (United States)^42^
H.5	Medicare payment for CCTA (facility)	$209.28	NA	Point estimate	2025 CMS Medicare Physician Fee Schedule (United States)^42^
H.6	Medicare payment for CCTA (interpretation)	$109.01	NA	Point estimate	2025 CMS Medicare Physician Fee Schedule (United States)^42^
H.7	Percentage of CCTAs that are same-day and show no obstructive CAD	50%	5	Author assumption	
H.8	Extra LOS from a same-day CCTA (hrs)	4.00	1.00	Author assumption	
H.9	Medicare payment for ETT (facility)	Bundled in APC 8011	NA	Point estimate	2025 CMS Medicare Physician Fee Schedule (United States)^42^
H.10	Medicare payment for ETT (interpretation + supervision)	$85.55	NA	Point estimate	2025 CMS Medicare Physician Fee Schedule (United States)^42^
H.11	Medicare payment for stress echo (facility)	Bundled in APC 8011	NA	Point estimate	2025 CMS Medicare Physician Fee Schedule (United States)^42^
H.12	Medicare payment for stress echo (interpretation)	$80.56	NA	Point estimate	2025 CMS Medicare Physician Fee Schedule (United States)^42^
I.1	Medicare reimbursement for uric acid	$4.52	NA	Point estimate	2025 CMS Medicare Physician Fee Schedule (United States)^42^
I.2	Medicare reimbursement for lipid panel	$13.39	NA	Point estimate	2025 CMS Medicare Physician Fee Schedule (United States)^42^
I.3	Medicare reimbursement for alanine aminotransferase	$5.30	NA	Point estimate	2025 CMS Medicare Physician Fee Schedule (United States)^42^
I.4	Medicare reimbursement for alkaline phosphatase	$5.18	NA	Point estimate	2025 CMS Medicare Physician Fee Schedule (United States)^42^
I.5	Medicare reimbursement for amylase	$6.48	NA	Point estimate	2025 CMS Medicare Physician Fee Schedule (United States)^42^
I.6	Medicare reimbursement for total protein	$3.67	NA	Point estimate	2025 CMS Medicare Physician Fee Schedule (United States)^42^
I.7	Medicare reimbursement for albumin	$4.95	NA	Point estimate	2025 CMS Medicare Physician Fee Schedule (United States)^42^
I.8	Medicare reimbursement for bilirubin	$5.02	NA	Point estimate	2025 CMS Medicare Physician Fee Schedule (United States)^42^
J.1	Observation facility cost (APC 8011)	$2,610.71	NA	Point estimate	2025 CMS Medicare Physician Fee Schedule (United States)^42^
J.2	Medicare facility cost for ED visit (CPT 99285)	$170.30	NA	Point estimate	2025 CMS Medicare Physician Fee Schedule (United States)^42^
**Bed hours saved**					
K	ED length of stay for patients with chest pain undergoing a traditional cardiac testing approach (bed-hours)	15.3	1.5	Normal	Retrospective data from 6 large EDs from 2013–2015 (United States)^43^
**Avoided hospitalizations**					
L	Percentage of ED observation chest pain visits where cardiac diagnostics are rate-limiting step to discharge	90%	NA	NA	Author assumption; see sensitivity analysis
**Average effective radiation dose from SPECT, PET, and CCTA (mSv)**					
M.1	Radiation associated with SPECT perfusion stress testing (mSv)	10	NA	NA	Dose Imaging Registry (United States)^44^–^46^
M.2	Radiation associated with PET perfusion stress testing (mSv)	3	NA	NA	Dose Imaging Registry (United States)^44^–^46^
M.3	Percentage of SPECT and PET that is male	57%	5.7%	Normal	Large international data registry in 2013 (International multicenter registry; 65 countries)^47^
M.4	Radiation associated with CCTA (mSv)	3	1	Normal	Dose Imaging Registry (United States)^44^–^46^
M.5	Percentage of CCTA that is male	47.3%	4.7%	Normal	Prospective study at 193 sites in North America between 2010–2013 (United States & Canada)^48^
N.1	Excess solid cancer cases per 100,000 persons from exposure to 100 mSv, males	800	400–1,600	BetaPERT	BEIR VII model (U.S.-adapted)^49^
N.2	Excess solid cancer cases per 100,000 persons from exposure to 100 mSv, females	1,300	690–2,500	BetaPERT	BEIR VII model (U.S.-adapted)^49^
N.3	Excess leukemia cases per 100,000 persons from exposure to 100 mSv, males	100	30–300	BetaPERT	BEIR VII model (U.S.-adapted)^49^
N.4	Excess leukemia cases per 100,000 persons from exposure to 100 mSv, females	70	20–250	BetaPERT	BEIR VII model (U.S.-adapted)^49^
N.5	Excess solid cancer deaths per 100,000 persons from exposure to 100 mSv, males	410	200–830	BetaPERT	BEIR VII model (U.S.-adapted)^49^
N.6	Excess solid cancer deaths per 100,000 persons from exposure to 100 mSv, females	610	300–1,200	BetaPERT	BEIR VII model (U.S.-adapted)^49^
N.7	Excess leukemia deaths per 100,000 persons from exposure to 100 mSv, males	70	20–220	BetaPERT	BEIR VII model (U.S.-adapted)^49^
N.8	Excess leukemia deaths per 100,000 persons from exposure to 100 mSv, females	50	10–190	BetaPERT	BEIR VII model (U.S.-adapted)^49^

*ADP*, Accelerated Diagnostic Protocols; *APC*, ambulatory payment classification; *BetaPERT*, Beta Program and Evaluation Review Technique; *CAD*, coronary artery disease; *CCTA*, coronary computed tomography angiogram; ; *CMS*, Centers for Medicare & Medicaid Services; *CPT*, current procedural terminology; *ED*, emergency department; *ETT*, exercise tolerance test; *hrs*, hours; *LOS*, length of stay; *mSv*, millisievert; *NA*, not applicable; *NHAMCS*, National Hospital Ambulatory Medical Care Survey; *PET*, positron emission tomography; *SD*, standard deviation; *SPECT*, single photon emission computed tomography; *US*, United States.

BetaPERT estimates use the mean as the most likely value, with the stated range for the minimum and maximum.

**Table 2:** Cardio Explorer price sensitivity analysis on primary outcome of cost savings.

Cardio Explorer Price Per Visit	National cost savings	30,000 annual adult visit ED cost savings	60,000 annual adult visit ED cost savings	90,000 annual adult visit ED cost savings
$0	$1.0 billion ($351 million)	$179,000 ($67,000)	$365,000 ($137,000)	$544,000 ($219,000)
$50	$675 million ($340 million)	$115,000 ($65,000)	$228,000 ($129,000)	$336,000 ($201,000)
$80	$596 million ($355 million)	$69,000 ($65,000)	$153,000 ($136,000)	$218,000 ($193,000)
$100	$357 million ($325 million)	$47,000 ($69,000)	$85,000 ($135,000)	$154,000 ($199,000)
$200	−$256 million* ($334 million)	−$90,000 ($65,000)	−$181,000 ($130,000)	−$268,000 ($200,000)

* Negative mean values represent net cost at that price.

*ED*, emergency department.

## References

[1] National Center for Health Statistics (2021). 2021 Emergency department summary data documentation: National Hospital Ambulatory Medical Care Survey (NHAMCS).

[2] Foy AJ, Liu G, Davidson WR (2015). Comparative effectiveness of diagnostic testing strategies in emergency department patients with chest pain. JAMA Intern Med.

[3] Skoien W (2016). Diagnostic yield of routine stress testing in low and intermediate risk chest pain patients under 40 years: a systematic review. Crit Pathw Cardiol.

[4] Yukselen Z, Majmundar V, Dasari M (2024). Chest pain risk stratification in the emergency department: current perspectives. Open Access Emerg Med.

[5] Backus BE, Six AJ, Kelder JC (2013). A prospective validation of the HEART score for chest pain patients at the emergency department. Int J Cardiol.

[6] Ashburn NP, Snavely AC, Paradee BE (2022). Age differences in the safety and effectiveness of the HEART Pathway accelerated diagnostic protocol for acute chest pain. J Am Geriatr Soc.

[7] Long B, Oliver J, Streitz M (2017). An end-user’s guide to the HEART score and pathway. Am J Emerg Med.

[8] Six AJ, Backus BE, Kelder JC (2008). Chest pain in the emergency room: value of the HEART score. Neth Heart J.

[9] Laureano-Phillips J, Robinson RD, Aryal S (2019). HEART score risk stratification of low-risk chest pain patients in the emergency department: a systematic review and meta-analysis. Ann Emerg Med.

[10] Ho AFW, Yau CE, Ho JS (2024). Predictors of major adverse cardiac events among patients with chest pain and low HEART score in the emergency department. Int J Cardiol.

[11] Stopyra JP, Riley RF, Hiestand BC (2019). The HEART Pathway randomized controlled trial one-year outcomes. Acad Emerg Med.

[12] Mahler SA, Riley RF, Hiestand BC (2015). The HEART Pathway randomized trial: identifying emergency department patients with acute chest pain for early discharge. Circ Cardiovasc Qual Outcomes.

[13] Veasey CJ, Snavely AC, Kearns ZL (2024). The high-sensitivity HEART Pathway safely reduces hospitalizations regardless of sex or race in a multisite prospective US cohort. Clin Cardiol.

[14] Halder D, Mathew R, Jamshed N (2021). Utility of HEART Pathway in Identifying low-risk chest pain in emergency department. J Emerg Med.

[15] Soares WE, Knee A, Gemme SR (2021). A prospective evaluation of clinical HEART score agreement, accuracy, and adherence in emergency department chest pain patients. Ann Emerg Med.

[16] Aung SSM, Roongsritong C (2022). A closer look at the HEART score. Cardiol Res.

[17] Arnett DK, Khera A, Blumenthal RS (2019). 2019 ACC/AHA guideline on the primary prevention of cardiovascular disease: part 1, lifestyle and behavioral factors. JAMA Cardiol.

[18] Pryor DB, Shaw L, McCants CB (1993). Value of the history and physical in identifying patients at increased risk for coronary artery disease. Ann Intern Med.

[19] Hamburger R, Spertus J, Winchester DE (2016). Utility of the Diamond-Forrester classification in stratifying acute chest pain in an academic chest pain center. Crit Pathw Cardiol.

[20] Diamond GA, Forrester JS (1979). Analysis of probability as an aid in the clinical diagnosis of coronary-artery disease. N Engl J Med.

[21] Wasfy MM, Brady TJ, Abbara S (2012). Comparison of the Diamond-Forrester method and Duke clinical score to predict obstructive coronary artery disease by computed tomographic angiography. Am J Cardiol.

[22] Baskaran L, Danad I, Gransar H (2019). A comparison of the updated Diamond-Forrester, CAD consortium, and CONFIRM history-based risk scores for predicting obstructive coronary artery disease in patients with stable chest pain: the SCOT-HEART coronary CTA cohort. JACC Cardiovasc Imaging.

[23] Fyyaz S, Rasoul H, Miles C (2021). ESC 2019 guidelines on chronic coronary syndromes: could calcium scoring improve detection of coronary artery disease in patients with low risk score. Findings from a retrospective cohort of patients in a district general hospital. JRSM Cardiovasc Dis.

[24] Vrints C, Andreotti F, Koskinas KC (2024). 2024 ESC Guidelines for the management of chronic coronary syndromes. Eur Heart J.

[25] Almeida J, Fonseca P, Dias T (2016). Comparison of coronary artery disease consortium 1 and 2 scores and Duke clinical score to predict obstructive coronary disease by invasive coronary angiography. Clin Cardiol.

[26] Baskaran L, Neo YP, Lee JK (2022). Evaluating the coronary artery disease consortium model and the coronary artery calcium score in predicting obstructive coronary artery disease in a symptomatic mixed Asian cohort. J Am Heart Assoc.

[27] McGinnis HD, Ashburn NP, Paradee BE (2022). Major adverse cardiac event rates in moderate-risk patients: does prior coronary disease matter?. Acad Emerg Med.

[28] Al’Aref SJ, Maliakal G, Singh G (2020). Machine learning of clinical variables and coronary artery calcium scoring for the prediction of obstructive coronary artery disease on coronary computed tomography angiography: analysis from the CONFIRM registry. Eur Heart J.

[29] Baskaran L, Ying X, Xu Z (2020). Machine learning insight into the role of imaging and clinical variables for the prediction of obstructive coronary artery disease and revascularization: an exploratory analysis of the CONSERVE study. PLoS One.

[30] Lee HG, Park SD, Bae JW (2023). Machine learning approaches that use clinical, laboratory, and electrocardiogram data enhance the prediction of obstructive coronary artery disease. Sci Rep.

[31] Eurlings CGMJ, Bektas S, Sanders-van Wijk S (2022). Use of artificial intelligence to assess the risk of coronary artery disease without additional (non-invasive) testing: validation in a low-risk to intermediate-risk outpatient clinic cohort. BMJ Open.

[32] Zellweger MJ, Tsirkin A, Vasilchenko V (2018). A new non-invasive diagnostic tool in coronary artery disease: artificial intelligence as an essential element of predictive, preventive, and personalized medicine. EPMA J.

[33] Harrison RL (2010). Introduction to Monte Carlo simulation. AIP Conf Proc.

[34] Baugh CW, Venkatesh AK, Hilton JA (2012). Making greater use of dedicated hospital observation units for many short-stay patients could save $3.1 billion a year. Health Aff (Millwood).

[35] Sechopoulos I, Rogers DWO, Bazalova-Carter M (2018). RECORDS: improved reporting of Monte Carlo radiation transport studies. Int J Radiat Oncol Biol Phys.

[36] Cairns C, Kang K (2022). National Hospital Ambulatory Medical Care Survey: 2022 emergency department summary tables.

[37] Yiadom MYAB, Baugh CW, McWade CM (2017). Performance of emergency department screening criteria for an early ECG to identify ST-segment elevation myocardial infarction. J Am Heart Assoc.

[38] Gottlieb M, Moyer E, Dissanayake V (2025). Disparities in troponin and ECG testing among emergency department patients with chest pain. Am J Emerg Med.

[39] Stopyra J, Snavely AC, Hiestand B (2020). Comparison of accelerated diagnostic pathways for acute chest pain risk stratification. Heart.

[40] Reeves RA, Halpern EJ, Rao VM (2021). Cardiac imaging trends from 2010 to 2019 in the Medicare population. Radiol Cardiothorac Imaging.

[41] Shaikh KA, Walters RW, Aboeata A (2022). Trends in utilization of coronary CT angiography in patients presenting with acute chest pain in United States: an analysis of the National Emergency Database. J Cardiovasc Comput Tomogr.

[42] Centers for Medicare & Medicaid Services (2025). 2025 CMS Medicare physician fee schedule.

[43] Hess EP, Hollander JE, Schaffer JT (2018). 2018 Testing a Decision Aid for Patients with Low-Risk Chest Pain in the Emergency Room – the Chest Pain Choice Trial.

[44] Einstein AJ (2012). Effects of radiation exposure from cardiac imaging: how good are the data?. J Am Coll Cardiol.

[45] Einstein AJ, Berman DS, Min JK (2014). Patient-centered imaging: shared decision making for cardiac imaging procedures with exposure to ionizing radiation. J Am Coll Cardiol.

[46] Fazel R, Gerber TC, Balter S (2014). Approaches to enhancing radiation safety in cardiovascular imaging: a scientific statement from the American Heart Association. Circulation.

[47] Shi L, Dorbala S, Paez D (2016). Gender differences in radiation dose from nuclear cardiology studies across the world: findings from the INCAPS registry. JACC Cardiovasc Imaging.

[48] Douglas PS, Hoffmann U, Lee KL (2014). Prospective multicenter imaging study for evaluation of chest pain: rationale and design of the PROMISE trial. Am Heart J.

[49] National Research Council (2006). Health risks from exposure to low levels of ionizing radiation: BEIR VII phase 2.

[50] Muennig P (2008). Cost-Effectiveness Analysis in Health: A Practical Approach.

[51] Qualls M, Pallin DJ, Schuur JD (2010). Parametric versus nonparametric statistical tests: the length of stay example. Acad Emerg Med.

[52] Law AM, Kelton WD (2007). Simulation Modeling and Analysis.

[53] Rees M (2015). Business Risk and Simulation Modelling in Practice: Using Excel, VBA And @RISK.

[54] Farrokhi M, Fallahian AH, Rahmani E (2025). Current applications, challenges, and future directions of artificial intelligence in emergency medicine: a narrative review. Arch Acad Emerg Med.

[55] Anand A, Shah ASV, Beshiri A (2019). Global adoption of high-sensitivity cardiac troponins and the universal definition of myocardial infarction. Clin Chem.

[56] Twerenbold R, Jaeger C, Rubini Gimenez M (2016). Impact of high-sensitivity cardiac troponin on use of coronary angiography, cardiac stress testing, and time to discharge in suspected acute myocardial infarction. Eur Heart J.

[57] Ganguli I, Cui J, Thakore N (2021). Downstream cascades of care following high-sensitivity troponin test implementation. J Am Coll Cardiol.

[58] Baugh CW, Blankstein R, Ganguli I (2023). Frequency, compliance, and yield of cardiac testing after high-sensitivity troponin accelerated diagnostic protocol implementation. Am J Emerg Med.

[59] Yau AA, Nguyendo LT, Lockett LL, Michaud E (2017). The HEART Pathway and hospital cost savings. Crit Pathw Cardiol.

[60] McCarthy C, Li S, Wang TY (2023). Implementation of high-sensitivity cardiac troponin assays in the United States. J Am Coll Cardiol.

[61] Lee HK, McCarthy CP, Jaffe AS (2025). High-sensitivity cardiac troponin assays: from implementation to resource utilization and cost effectiveness. J Appl Lab Med.

[62] Ashburn NP, McCord JK, Snavely AC (2024). Navigating the observation zone: do risk scores help stratify patients with indeterminate high-sensitivity cardiac troponins?. Circulation.

[63] Hollander JE, Than M, Mueller C (2016). State-of-the-art evaluation of emergency department patients presenting with potential acute coronary syndromes. Circulation.

[64] Pines JM, Batt RJ, Hilton JA (2011). The financial consequences of lost demand and reducing boarding in hospital emergency departments. Ann Emerg Med.

[65] Foley M, Kifaieh N, Mallon WK (2011). Financial impact of emergency department crowding. West J Emerg Med.

[66] Than M, Herbert M, Flaws D (2013). What is an acceptable risk of major adverse cardiac event in chest pain patients soon after discharge from the emergency department? A clinical survey. Int J Cardiol.

[67] Abul-Husn NS, Kenny EE (2019). Personalized medicine and the power of electronic health records. Cell.

[68] Tan A, Durbin M, Chung FR (2020). Design and implementation of a clinical decision support tool for primary palliative care for emergency medicine (PRIM-ER). BMC Med Inform Decis Mak.

[69] Vinson DR, Warton EM, Durant EJ (2024). Decision support intervention and anticoagulation for emergency department atrial fibrillation: the O’CAFÉ stepped-wedge cluster randomized clinical trial. JAMA Netw Open.

[70] Loupas MA, Athanasakis K, Zavras D (2026). Willingness to pay per QALY: a systematic review of demand-side valuations with a focus on age and disease severity. Appl Health Econ Health Policy.

[71] Schultz CH, Fairley R, Murphy LSL, Doss M (2020). The risk of cancer from CT scans and other sources of low-dose radiation: a critical appraisal of methodologic quality. Prehosp Disaster Med.

[72] Baugh CW, Bohan JS (2008). Estimating observation unit profitability with options modeling. Acad Emerg Med.

[73] Jacob V, Thota AB, Chattopadhyay SK (2017). Cost and economic benefit of clinical decision support systems for cardiovascular disease prevention: a community guide systematic review. J Am Med Inform Assoc.

