# Supplementary material for: Artificial Intelligence-Driven Cost Savings from Emergency Department Chest Pain Patient Evaluation: A Monte Carlo Simulation
Source: West J Emerg Med. 2026 Jul 28;27(5):1340–52. doi: 10.5811/westjem.56973 (PMC13660611; doi:10.5811/westjem.56973)
Supplement: Supplementary file 1 [file wjem-27-1340-s001.docx]

**Online Supplement**

**Table 1:** Medication Use Considered by Cardio Explorer

| **Variable** | **Descriptions** | **Justification** | **Drug Classes** | **Validity interval** |
| --- | --- | --- | --- | --- |
| Lipid-lowering therapy | Use of statins or non-statin LDL-lowering agents. | Lipid-lowering therapy is a cornerstone in reducing cardiovascular risk in CCS. [ESC Guidelines, 2024] | Statins, Ezetimibe, PCSK9 inhibitors, Bempedoic acid, Inclisiran | Last 3 months |
| RAAS-inhibiting therapy | ACE inhibitor or ARB. | RAAS inhibitors reduce cardiovascular events in hypertension, HF, and post-MI patients. [ESC Guidelines, 2024 | ACE inhibitors, ARBs | Last 3 months |
| Beta-blocker therapy | Any beta blocker. | Beta blockers reduce myocardial oxygen demand and improve symptoms in CCS. [ESC Guidelines, 2024] | Beta blockers | Last 3 months |
| Calcium channel blocker therapy | Any calcium channel blocker. | Calcium channel blockers relieve angina and control heart rate or blood pressure. [ESC Guidelines, 2024] | Dihydropyridines (DHP), Non-DHP (rate-limiting) | Last 3 months |
| Diuretic therapy | Any diuretic. | Diuretics manage blood pressure and symptoms of heart failure or volume overload. [ESC Guidelines, 2024] | Thiazides, Loop diuretics, MRAs | Last 3 months |
| Anti-anginal nitrate therapy | Nitrates for angina. | Nitrates provide rapid relief of angina by vasodilation and reducing myocardial oxygen demand. [ESC Guidelines, 2024] | Organic nitrates | Last 3 months |
| Antiplatelet therapy | Any antiplatelet agent. | Antiplatelet agents reduce the risk of thrombotic cardiovascular events. [ESC Guidelines, 2024] | Aspirin and P2Y12 inhibitors | Last 3 months |
| Glucose-lowering therapy | Any glucose-lowering medication. | Glucose-lowering therapy reduces cardiovascular complications in diabetes. [ESC Guidelines, 2024] | Insulin, Metformin, SGLT2 inhibitors, GLP-1 RAs, Sulfonylureas, DPP4 inhibitors | Last 3 months |

**Table 2:** RECORDS: Reporting Monte Carlo studies

| Section and topic | No | Item | Inclusion |
| --- | --- | --- | --- |
| Title | 1 | Identification as a Monte Carlo study | 🗸 |
| Methods |  |  |  |
| Software | 2 | Name of Monte Carlo software used, including a reference to a paper and/or report describing the package, if available. If the selected code is not commonly used for the application of the current study or if it necessitated considerable modification, then justification for why a certain Monte Carlo code was selected | 🗸 |
|  | 3 | Release number and/or release date of Monte Carlo software used | 🗸 |
|  | 4a | If the code has been previously validated or is being validated against previous publications, description of the code’s validation in similar or at least relevant applications, preferably via references | 🗸 |
|  | 4b | If code is being validated against experimental measurements, then detailed description of experimental conditions and of simulation study. For the latter, include any assumptions and simplifications of the experimental condition | Not applicable |
| Hardware | 5a | If study is related to Monte Carlo efficiency, specification of CPU/GPU time, compiler information, and system used to perform the simulations, including CPU/GPU model number | Not applicable |
|  | 5b | Otherwise specification of order of magnitude of CPU/GPU time and CPU/GPU model number | Not applicable |
| Geometry | 6 | Description of simulation geometry, using drawings and tables as needed. Provide all relevant dimensions which are not proprietary and non-disclosable. If possible, provide equivalent composition and dimension information in place of non-disclosable proprietary details | Not applicable |
| Materials | 7 | Description of material composition and mass density of each item in the geometry, with references if applicable. Provide the elemental composition and/or, if applicable, the Hounsfield Units conversion method | Not applicable |
| Source | 8 | Description of the source, including: source of phase-space files if used; model used to generate source; and model parameter values. Provide reference, if applicable | Not applicable |
| Physics and transport | 9 | Specification of the cross-section data used along with derived quantities such as stopping powers | Not applicable |
|  | 10 | Specification of relevant transport parameters used, such as energy and particle weight cutoffs, step sizes and thresholds. Also transport algorithms if there is more than one option for the code used. Specifying use of the default is adequate if it is unique and documented by citation | Not applicable |
|  | 11 | Specification of variance reduction (VRT) and approximate efficiency improvement techniques (AEIT) used, and their parameters. References describing them or specific descriptions of any new techniques should be included if they are not included in the Monte Carlo software as built-in, documented options | Not applicable |
| Scoring | 12 | Specification of the relevant scored quantities. Provide tally names if using a standard Monte Carlo software that includes them, but also include what physical quantity is scored by the tally. When results are binned, tabulated values should make clear what the variable means, e.g., the mid-point, top or range of a bin | Not applicable |
|  | 13 | Number of histories and number of source particles used. If various simulations involve different numbers of histories and source particles, at least provide range | Not applicable |
|  | 14 | Description and references, as appropriate, of the method used to estimate statistical uncertainty, including if estimated by the history-by-history or the batch method | Not applicable |
| Analysis | 15 | Description of how scored quantities are normalized and/or converted to other metrics. List physical conversion factors used and provide references, if applicable, if there are multiple values for these in the literature | Not applicable |
|  | 16 | Description of how scored quantities are de-noised or otherwise filtered, with references, if applicable. If none, then this should be specifically mentioned | Not applicable |
| Results | 17 | Results of validation, unless code has been previously validated | Not applicable |
|  | 18 | Scored quantities with statistical uncertainty including a specification of the confidence limits used. In general, graphical representation of results such as depth-dose curves or spectra should be histogram rather than point plots, and include uncertainty estimates with error bars in the graphs or text in the caption | Not applicable |
| Discussion | 19 | Discussion of study limitations, including sources of potential bias, statistical uncertainty, and generalizability. | 🗸 |
|  | 20 | Discussion of assumptions and approximations and their potential effect on the results, given the knowledge gained. | 🗸 |

Adapted from: Sechopoulos I, Rogers DWO, Bazalova-Carter M, Bolch WE, Heath EC, McNitt-Gray MF, Sempau J, Williamson JF. RECORDS: improved Reporting of montE CarlO RaDiation transport Studies. Int J Radiat Oncol Biol Phys. 2018 Jul 15;101(4):792-793.
